# NF-Y subunits overexpression in gastric adenocarcinomas (STAD)

**DOI:** 10.1038/s41598-021-03027-y

**Published:** 2021-12-09

**Authors:** Alberto Gallo, Mirko Ronzio, Eugenia Bezzecchi, Roberto Mantovani, Diletta Dolfini

**Affiliations:** Dipartimento di Bioscienze, Università degli Studi di Milano, Via Celoria 26, 20133 Milan, Italy

**Keywords:** Cancer, Computational biology and bioinformatics

## Abstract

NF-Y is a pioneer transcription factor—TF—formed by the Histone-like NF-YB/NF-YC subunits and the regulatory NF-YA. It binds to the CCAAT box, an element enriched in promoters of genes overexpressed in many types of cancer. NF-YA is present in two major isoforms—NF-YAs and NF-YAl—due to alternative splicing, overexpressed in epithelial tumors. Here we analyzed NF-Y expression in stomach adenocarcinomas (STAD). We completed the partitioning of all TCGA tumor samples (450) according to molecular subtypes proposed by TCGA and ACRG, using the deep learning tool DeepCC. We analyzed differentially expressed genes—DEG—for enriched pathways and TFs binding sites in promoters. CCAAT is the predominant element only in the core group of genes upregulated in all subtypes, with cell-cycle gene signatures. NF-Y subunits are overexpressed, particularly NF-YA. NF-YAs is predominant in CIN, MSI and EBV TCGA subtypes, NF-YAl is higher in GS and in the ACRG EMT subtypes. Moreover, NF-YAl^high^ tumors correlate with a discrete Claudin^low^ cohort. Elevated NF-YB levels are protective in MSS;TP53^+^ patients, whereas high NF-YAl/NF-YAs ratios correlate with worse prognosis. We conclude that NF-Y isoforms are associated to clinically relevant features of gastric cancer.

## Introduction

Gastroesophageal tumors are among the most widespread cancers worldwide^1^. Stomach adenocarcinomas—STAD—share a survival outcome of patients that, despite many efforts, remains poor. The Lauren histological classification divides gastric cancers into intestinal (IT), diffuse (DF) and mixed (MX)^2,3^. Further microarrays profilings studies have since classified tumors according to molecular subtypes^4–7^. More recently, TCGA has proposed a classification based on genetic mutations, chromosomal alterations, epigenetic features and RNA-seq expression data that included four subtypes: EBV (EBV-infected), MSI (MicroSatellite Instability), GS (Genomically Stable) and CIN (Chromosomal Instability)^8^. In parallel, the ACRG (Asian Cancer Research Group) proposed another classification, originally based on independent microarray profilings, also consisting of four subtypes: EMT (Epithelial to Mesenchymal Transition), MSS;TP53^-^ (MicroSatellite Stable, inactive tumor protein 53), MSS;TP53^+^ and MSI^9,10^. The two classifications are partially overlapping (Reviewed in Refs.^11–13^).

In general, cellular transformation causes—and in some cases is caused by—changes in mRNA production patterns. The first step in this process is the binding of sequence-specific transcription factors—TFs—to DNA elements in promoters and enhancers, entailing recruitment of chromatin modifying Cofactors^14^. Changes in the structure or expression of TFs can cause permanent changes that lead to transformation. The identification of TFBSs—transcription factor binding sites—in promoters of genes overexpressed in cancer led to the identification of the CCAAT box as one of the most widely enriched^15^. CCAAT is typically crucial for high-level expression of genes^16^. This box is recognized by NF-Y, a heterotrimer formed by the histone fold domain—HFD—dimer NF-YB/NF-YC and the sequence-specific NF-YA^17^. NF-YA has two alternatively spliced isoforms—NF-YAs and NF-YAl—differing in 28/29 amino acids coded by exon 3^18^. NF-YC is also present in multiple isoforms, resulting from alternative splicing at the C-terminal of the protein^19^. In both subunits, this involves the glutamine-rich trans-activation domains (TADs), while the subunits-interaction and DNA-binding domains are common to all isoforms.

NF-Y subunits are rarely mutated in tumors, yet the NF-Y regulome—ChIP-seq and functional analysis—point to cell-cycle and metabolic pathways being positively affected^20^: specifically, rate-limiting, cancer-promoting genes of different anabolic routes—amino acids, lipids, nucleotides—are activated^21^.

Reports on the expression of NF-Y subunits in tumors emerged recently. In ovarian^22,23^, breast^24,25^, lung^26,27^, liver^28^ and head and neck squamous cell carcinomas (HNSCC)^29^, overexpression of NF-YA was reported. As for gastric cancer, two studies provide evidence for a specific function of NF-YA: microarray-based differentially expressed genes (DEG) of gastric cancer identified NF-YA as a key TF, specifically in the DF subtype, with prognostic significance^30^; NF-YA inactivation has a more profound growth suppressive effect in a DF than in a IT cell line. Another study analyzing TCGA data found high expression of NF-YA, including of the protein in STAD specimens^31^; this correlated with Cyclin E, a gene often amplified and overexpressed in STAD datasets^32,33^. These two studies did not report on the relative levels of the two major NF-YA subunits, which are clinically important in breast, lung and HNSCC cancers^25–27^, nor of the HFD subunits, which might be relevant in light on our recent finding on their overexpression in liver Hepatocarcinomas and HNSCC^28,29^. We report here on the analysis of STAD RNA-seq data present in TCGA, as further classified according to TCGA and ACRG. We confirm NF-YA global overexpression, extend this finding to HFD subunits, and investigate the isoforms of NF-YA.

## Results

### NF-Y subunits are overexpressed in STAD

**Figure 1:**
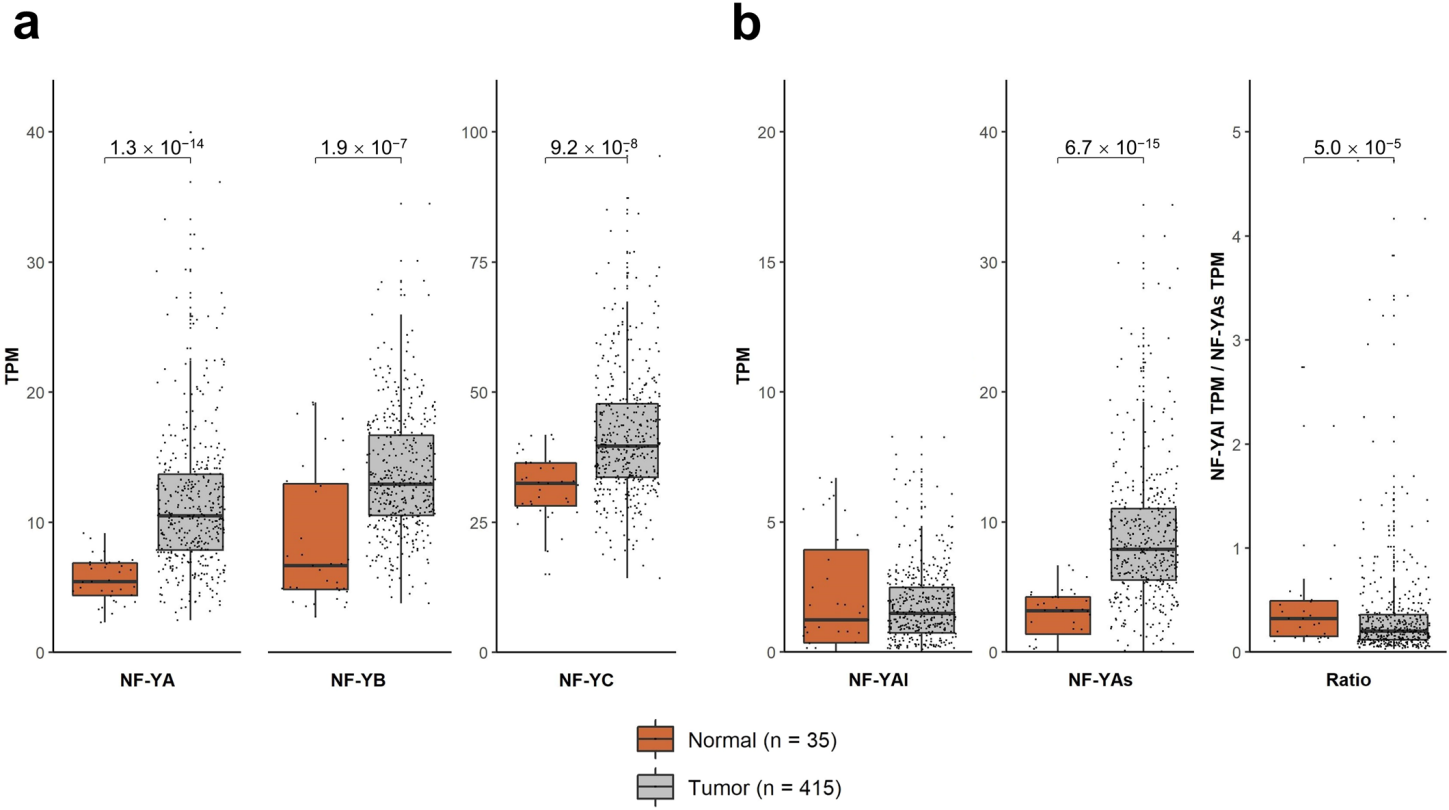
NF-Y subunits are overexpressed in STAD. (**a**) Box plots of expression levels of the three NF-Y subunits at gene level in the TCGA-STAD dataset, measured in TPMs. (**b**) Same as (**a**) with the analysis of the NF-YA, -NF-YAs, NF-YAl-isoforms levels as well as the NF-YAl/NF-YAs ratio. p values are calculated using the Wilcoxon rank-sum test.

Inspection of NF-Y subunits expression of the TCGA datasets (http://firebrowse.org) suggested that expression of NF-YA is globally increased in epithelial tumors^25^. We downloaded the available STAD RNA-seq dataset^8^ and analyzed NF-Y subunits: NF-YA is robustly increased in STAD (p value: 10^–14^). NF-YB and NF-YC are also increased (p values: 10^–07/08^) (Fig. 1a). We then analyzed the levels of NF-YA isoforms: Fig. 1b shows that the levels of the “short” NF-YAs increase in tumors (p value 10^–15^), unlike NF-YAl. In conclusion, we confirm a generalized overexpression of NF-Y subunits, especially NF-YA, in STAD.

The predominance of NF-YAs prompted us to verify the relative expression in gastric cancer cell lines. For this, we interrogated two repositories: the Broad Institute CCLE—Cancer Cell Lines Encyclopedia (https://portals.broadinstitute.org/ccle/about) and a recently described set of gastric cancer lines^34^; overall, we analyzed 50 cell lines, with a partial overlap of lines common to the two datasets. We downloaded RNA-seq data, mapped reads and analyzed NF-Y subunits levels. The results are shown in Fig. S1: the overall levels of NF-YA mRNA expression are variable with the majority, but not all, cell lines expressing primarily NF-YAs (Fig. S1a). The levels of the two HFD subunits, particularly NF-YB, are comparably less variable among the cell lines (Fig. S1b,c). We conclude that NF-Y subunits are overexpressed in STAD, particularly NF-YA, whose predominant isoform is NF-YAs, in gastric tumors and cell lines.

### Expression of NF-Y isoforms in STAD subtypes

**Figure 2:**
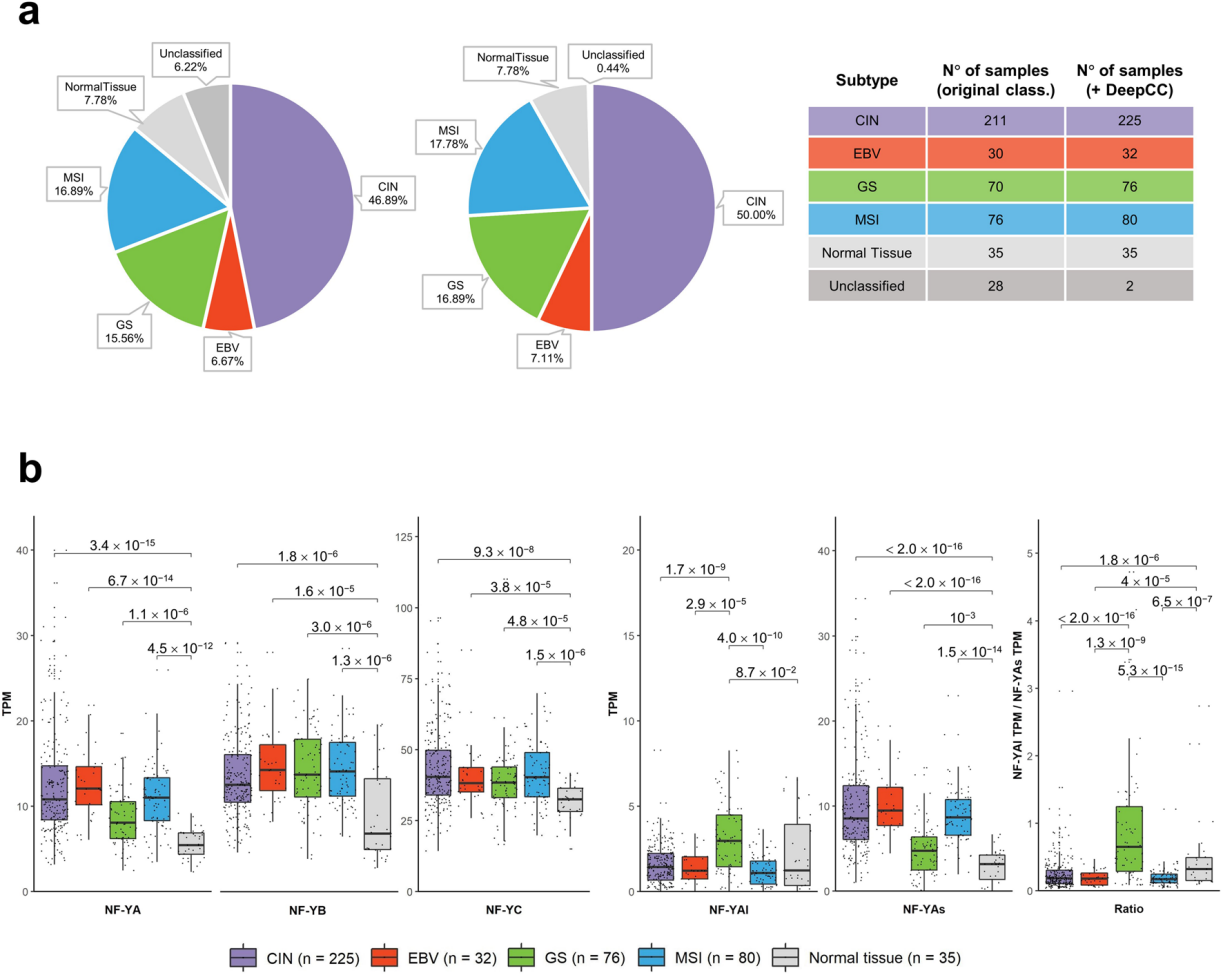
NF-Y levels in four subtypes of STAD (TCGA classification). (**a**) Classification of all TCGA samples for which there are RNA-seq data in the four subtypes. In the left pie the currently available samples (Unclassified 6.22%), in the right one our complete classification. The numbers for each subtype are in the scheme to the right. (**b**) Box plots of expression levels of the three NF-Y subunits at gene level (Left Panels), as well as of NF-YAl, NF-YAs and the NF-YAl/NF-YAs ratio (Right Panels), in the four subtypes of the TCGA STAD dataset as well as in normal tissues, measured in TPMs. p values are calculated using the Wilcoxon rank-sum test.

According to several genetic, epigenetic and functional parameters, TCGA classified STAD in four subtypes^8^. Since overexpression of NF-Y subunits could be limited to one -or more- of the subtypes, we investigated the levels of the three subunits in the four cohorts. Currently, RNA-seq data on 415 tumors are available, of which 387 were categorized by TCGA. We first classified all tumors for which there are RNA-seq data, employing the DeepCC machine learning tool^35^, with a training set represented by those already classified by TCGA: the relative proportions are indeed essentially maintained (Fig. 2a). Figure 2b (Left Panels) shows that the relative increase of NF-YA is similar in CIN, EBV and MSI (p values of 10^–12/15^ relative to normal samples), but in GS, the levels are lower. NF-YB and NF-YC are increased at comparable levels in all subtypes.

As for the isoforms, the data are shown in Fig. 2b (Right Panels): NF-YAs is increased in MSI, EBV and CIN (p values 10^–14/16^ with respect to normal samples), less in GS. NF-YAl, instead, shows a significant increase in GS. As a consequence of these changes, the NF-YAl/NF-YAs ratio is substantially increased in GS with respect to the other subtypes. In summary, overexpression of NF-YAs is generally widespread, but there is a distinctly higher NF-YAl/NF-YAs ratio in GS tumors.

### STAD differentially expressed genes—DEG—have CCAAT in promoters

To gain insight on the gene expression programs altered in STAD, we compared RNA-seq data of STAD tumors to those of the respective normal samples, using a |log_2_FC|> 0.5, FDR < 0.01 threshold. The lists of DEG are in Supplementary Table S1. We analyzed the promoters (− 450 to + 50 from the TSS) of overexpressed genes with the Pscan software, which pinpoints enriched TFs matrices^36^. The NF-Y matrix is absent, and E2Fs and SP/KLFs are at the top of the list of upregulated genes (Fig. S2a, Left Panel). As for downregulated genes (Fig. S2a, Right Panel), CCAAT is absent, and Zn Fingers TFs are enriched. Thereafter, we used KOBAS to identify Gene Ontology terms in DEG: in upregulated genes, nuclear terms—*nucleolus*, *nuclear chromatin*, *cell division*, *DNA replication*—predominate; different terms are also present in downregulated genes (Fig. S2b).

**Figure 3:**
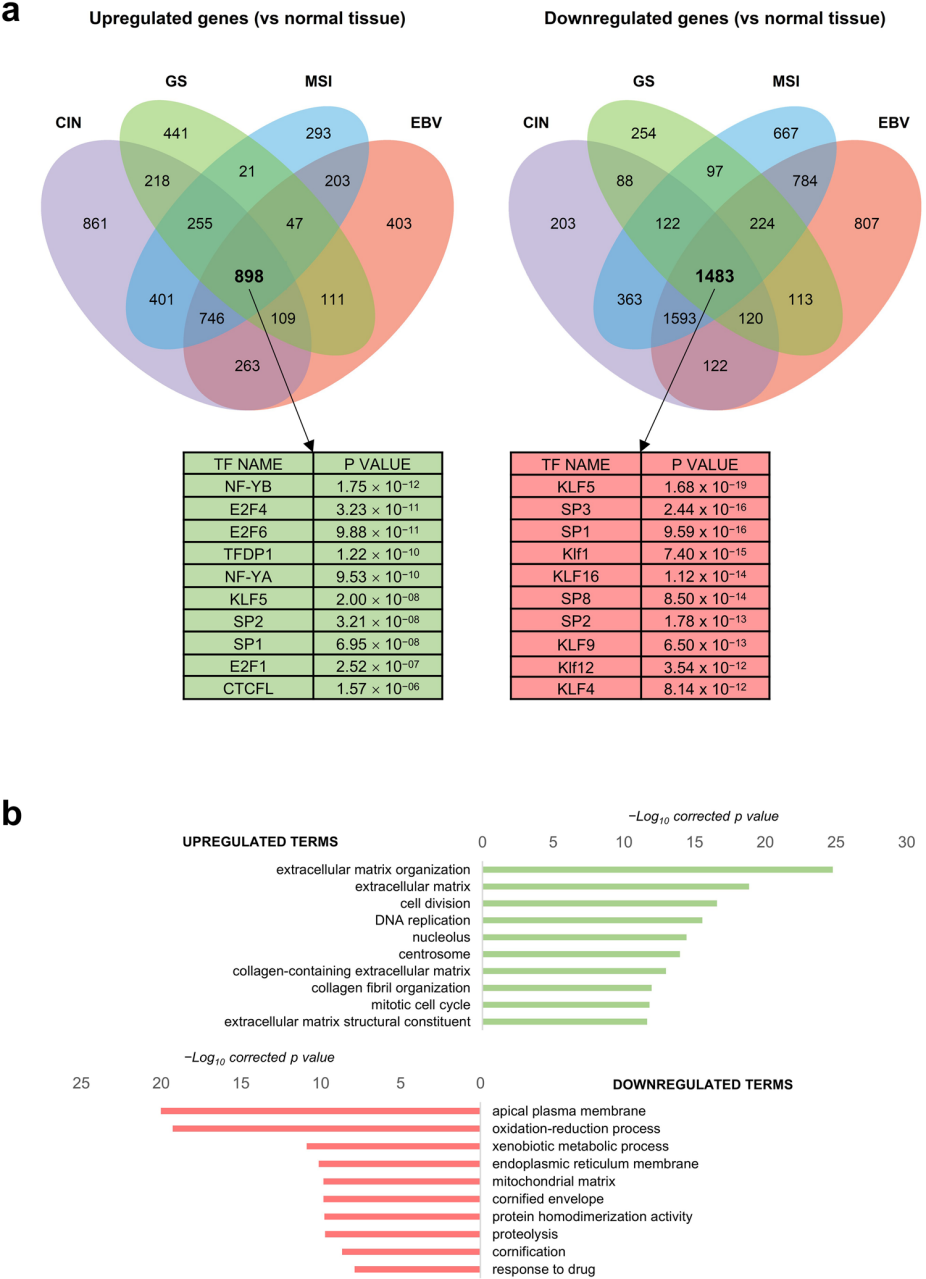
DEG in STAD core. (**a**) Upper Panels, Venn diagrams of DEG in the four TCGA subtypes. Left Diagram, upregulated genes, Right Diagram, downregulated genes. In the lower Panels, we show the Pscan analysis of Transcription Factors Core. (**b**) KOBAS analysis of upregulated and downregulated GO terms in the core set of commonly deregulated genes in the four STAD subtypes.

With the same thresholds, we then performed analysis of RNA-seq of the individual TCGA subtypes. Venn diagrams of the overlaps are shown in Fig. 3a and the lists of genes are in Supplementary Table S2. As for subtype-specific TFBS, distinct matrices are enriched in the four subtypes (Fig. S3a): SP1/2 in CIN, ETS-family in EBV, Zn fingers TFs in GS and MSI (EGR1/2/3, Sp2/4). We analyzed Gene Ontology terms of DEG: Fig. S3b shows specific gene signatures for individual subtypes: in CIN, *cellular protein metabolism, spermatogenesis*; in EBV, *viral process*, *T cell signaling*; in GS, *extracellular matrix*, *cell adhesion*; in MSI, *nucleolus*. Analysis of the common set of 898 genes upregulated in all subtypes have NF-Y at the top of the enriched matrices, and features described in global DEG, such as *extracellular matrix*, *cell division*, *DNA replication*, with the addition of *extracellular matrix* terms (Fig. 3b). Overall, we conclude that CCAAT is the primary site only in promoters of commonly upregulated genes, but it is absent in those specific to each TCGA subtype.

### Clinical outcome of NF-Y overexpression in STAD according to the TCGA subtypes

**Figure 4:**
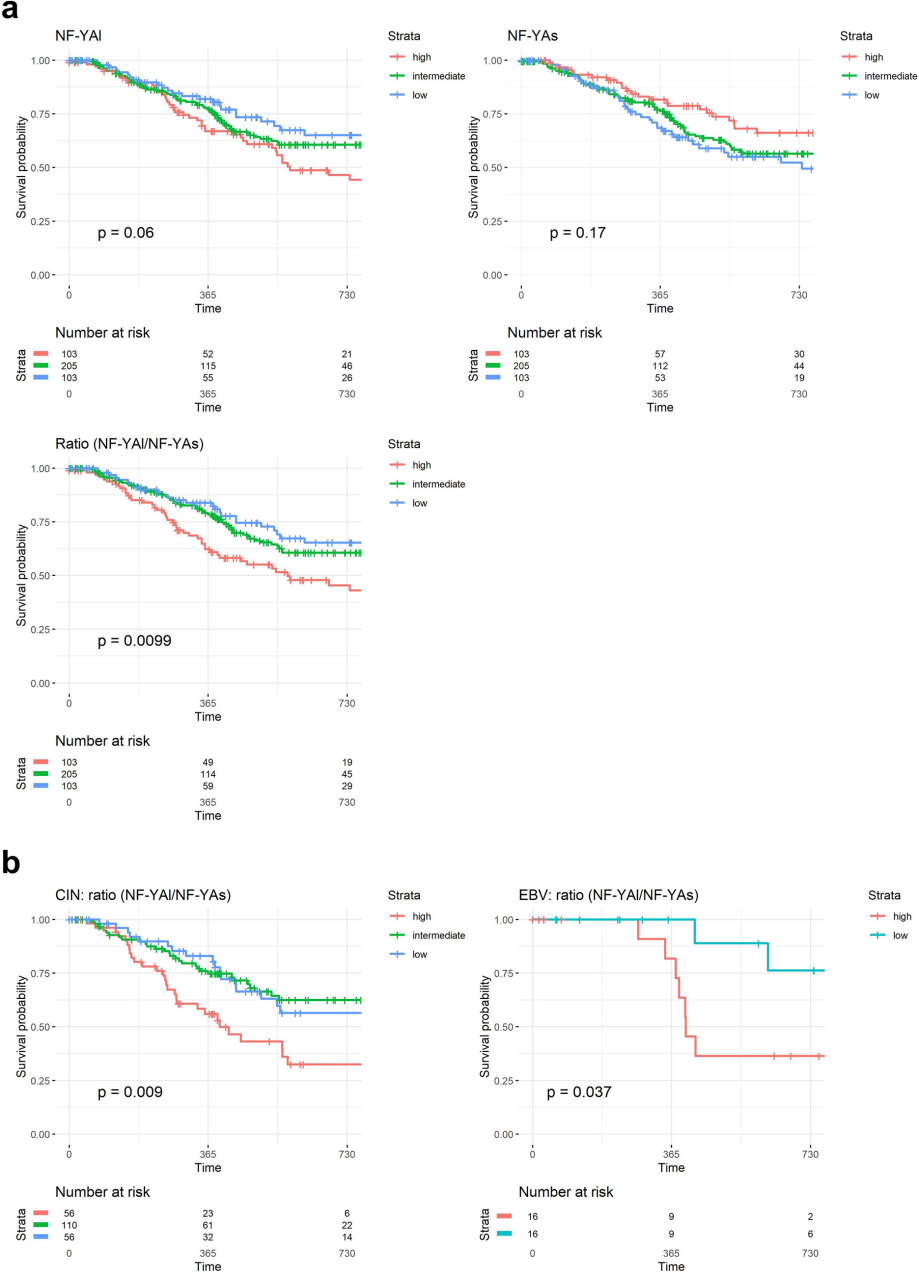
Clinical outcome of NF-Y expression according to the TCGA classification. (**a**) Upper Panels, Progression-Free-Interval-PFI-curves of survival probability of STAD tumors with stratification according to quartiles of NF-YA long and short isoforms (Intermediate, High and Low). PFI curves of NF-YAl/NF-YAs ratios are shown in the Lower Panel. We stratified all available tumors in the three groups according to the TPM levels: Low = first quartile, high = fourth quartile and intermediate for values included in the two middle quartiles. p values are calculated using the log-rank test. (**b**) PFI curves of the NF-YAl/NF-YAs ratios in the CIN (Left Panel) and EBV tumors (Right Panel). In this latter case, we stratified samples in two bins, High (third and fourth quartiles) and Low (first and second quartiles).

We stratified the progression free interval—PFI—of STAD patients according to High, Intermediate, Low levels of NF-Y subunits expression. In addition, we considered the ratios of NF-YAl/NF-YAs, because this parameter was more informative than the overall levels of the two isoforms to predict patient outcomes in breast, lung and HNSCC cancers^25–27,29^. No correlation is scored according to the different levels of NF-YA and of the HFD subunits (Fig. S4), nor to the ones of NF-YAl and NF-YAs isoforms (Fig. 4a, Upper Panels). As for the NF-YAl/NF-YAs ratios, instead, we did find a robust correlation with worse prognosis (p value 0.0099) (Fig. 4a, Lower Panel). We then focused on PFIs of NF-YA ratios stratified according to the single subtypes: a correlation with poor prognosis was scored in CIN and EBV (Fig. 4b), but not in GS and MSI (Fig. S5). In summary, a higher NF-YAl/NF-YAs ratio does have relevant clinical implication in STAD, globally and in specific TCGA subtypes.

### Expression of NF-Y according to the ACRG classification

**Figure 5:**
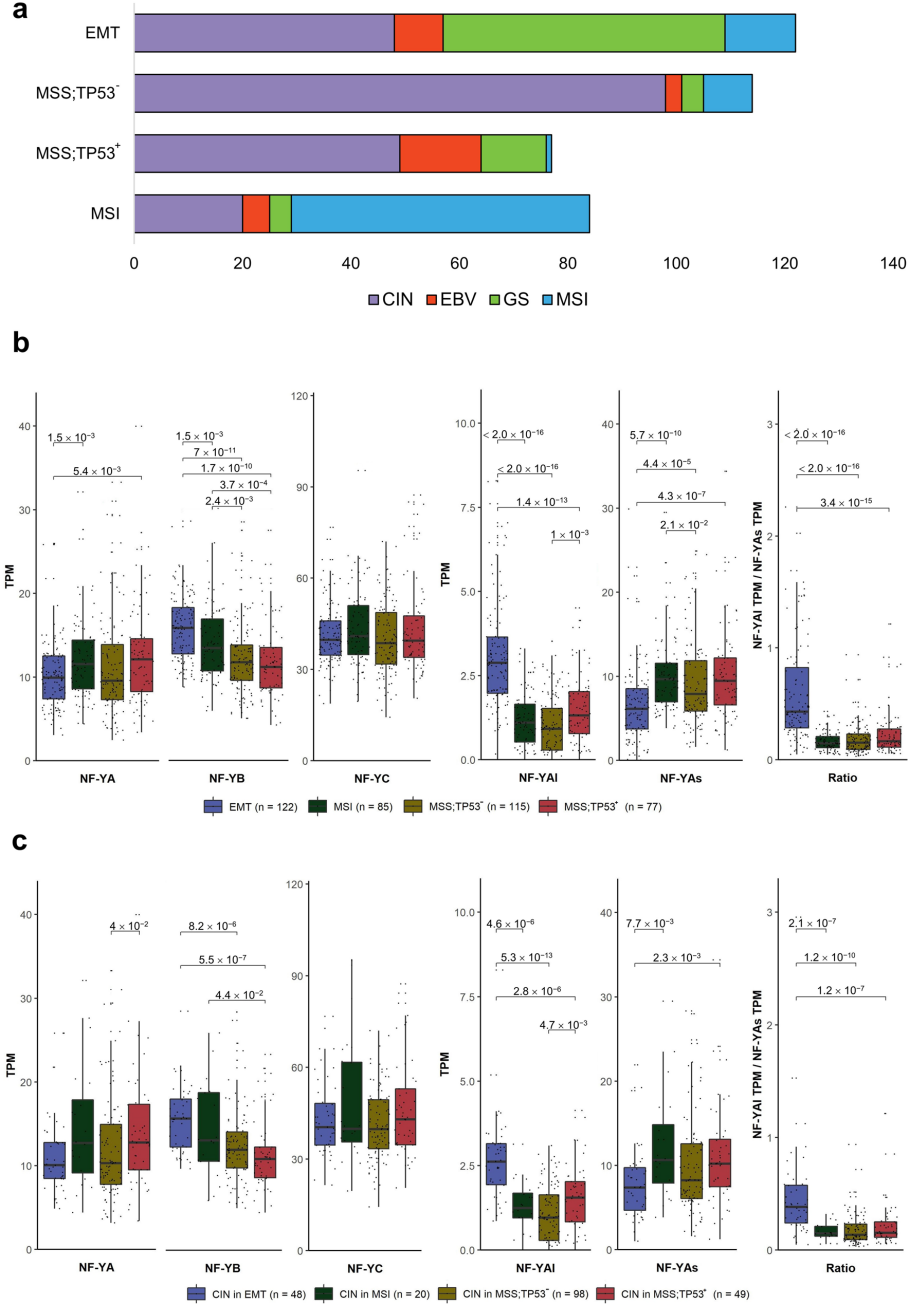
NF-Y expression in the ACRG subtypes. (**a**) Partitioning of TCGA subtypes in the four ACRG subtypes, according to the classification of TCGA RNA-seq dataset. (**b**) Box plot analysis of NF-Y subunits (Left Panel) and NF-YA isoforms (Right Panel) in the four ACRG subtypes. (**c**) Same as (**b**), except that expression was computed in the TCGA CIN samples partitioned in the ACRG subtypes. p values are calculated using the Wilcoxon rank-sum test.

A second STAD molecular classification was proposed by ACRG. This was originally based on profiling analysis, and thereafter applied to the TCGA RNA-seq database on a partial set of 204 samples^9^. As above, we first used DeepCC and the training set to classify all TCGA tumors in the four ACRG subclasses: unclassified samples are reduced from 211 to 16 (Fig. S6a). The proportion of the four classes are relatively well maintained, with EMT being the most abundant (122 samples). A direct comparison between the TCGA and ACRG classifications is shown in Fig. 5a: most GS samples are found in EMT, which also harbors a sizeable number of CIN; MSI samples are largely shared, while EBV are partitioned among the four subclasses. With the extended ACRG dataset on hand, we evaluated the levels of NF-Y subunits and isoforms: Fig. 5b (Left Panels) shows similar levels of NF-YA and NF-YC, lower levels of NF-YB in MSS;TP53^-^ and MSS;TP53^+^. Figure 5b (Right Panels) shows higher levels of NF-YAl, and lower of NF-YAs, in EMT samples, leading to an increased ratio of these isoforms. The presence of CIN samples in all ACRG subtypes, particularly EMT, led us to analyze NF-Y expression of CIN within ACRG subclasses: globally, the levels are similar (Fig. 5c, Left Panels), with those within the EMT group having distinctly higher levels of NF-YAl, lower NF-YAs and, by consequence, higher ratios (Fig. 5c, Right Panels). Note that analysis of STAD cell lines shows that most EMT lines, classified as such by Lee et al. ^34^, indeed express the lowest levels of NF-YAs and highest of NF-YAl (Fig. S1a). We conclude that the EMT subclass of ACRG includes GS, as well as a portion of tumors catalogued as CIN, having a high ratio between NF-YAl and NF-YAs.

### Clinical outcome of NF-Y expression according to the ACRG subtypes

**Figure 6:**
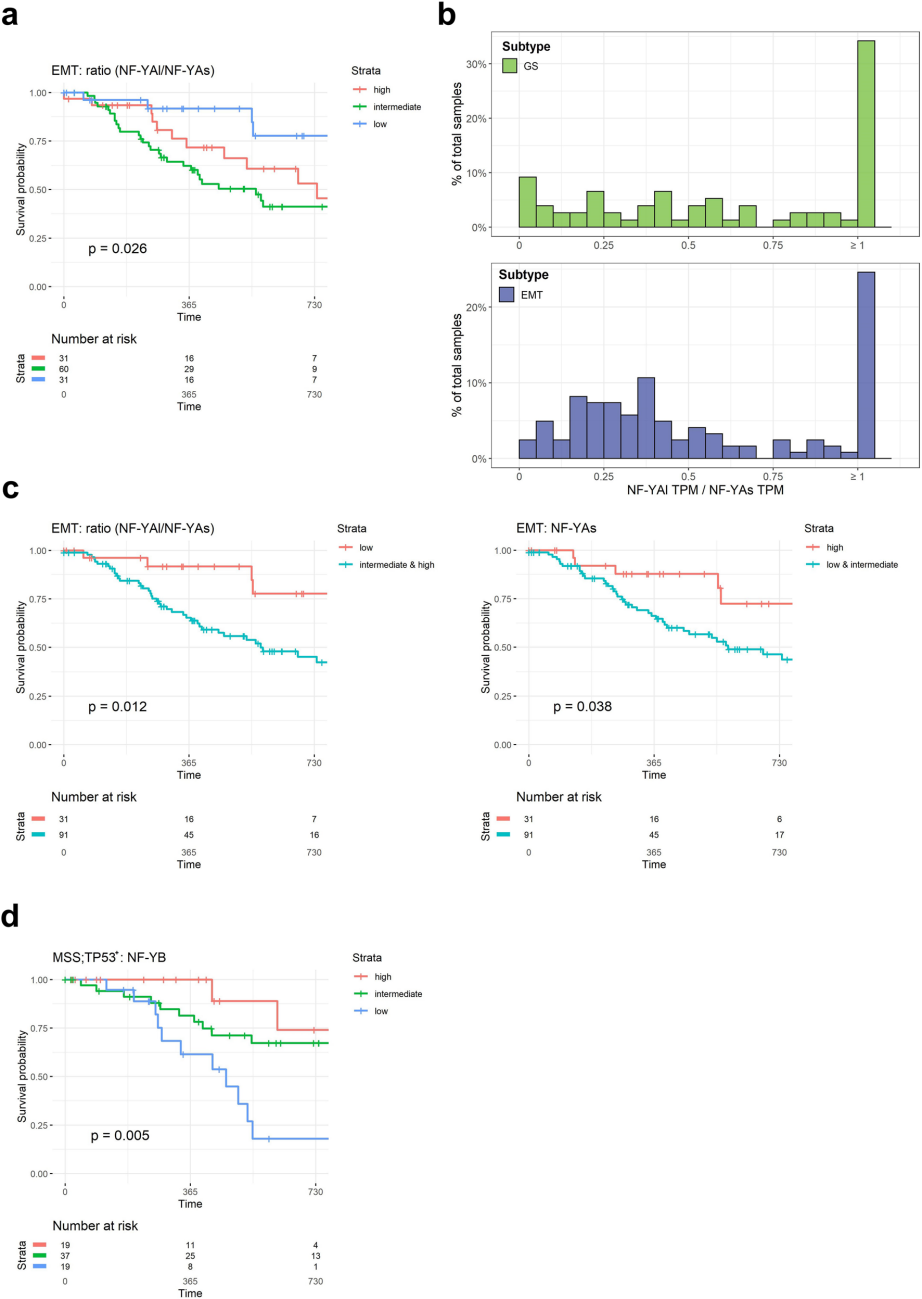
Clinical outcome of NF-Y expression according to the ACRG classification. (**a**) Progression-Free-Interval curves of survival probability of EMT tumors with stratification according to quartiles of NF-YAl/NF-YAs ratio (Intermediate, High and Low). We stratified all available tumors in the three groups according to the ratio levels: Low = first quartile, High = fourth quartile, and Intermediate for values included in the two middle quartiles. p values are calculated using the log-rank test. (**b**) Distribution of NF-YAl/NF-YAs ratios in the TCGA GS (Upper Panel) and ACRG EMT (Lower Panel) samples. The width of each bin was set to 0.05, and ratios equal to or greater than one were included in the last bin. (**c**) PFI curves in EMT tumors of NF-YAl/NF-YAs (Left Panel) and NF-YAs (Right Panel) stratified in Low and Intermediate/High values or High and Intermediate/Low, respectively. (**d**) Same as (**a**), except that the levels of NF-YB expression were correlated to prognosis in MSS;TP53^+^ tumors.

Next, we evaluated the clinical outcome of patients according to the ACRG classification. Stratification according to NF-YAl/NF-YAs ratios indicate no clinical relevance in MSI, MSS;TP53^−^ and MSS;TP53^+^, but worst prognosis with high and intermediate levels in EMT (Fig. 6a). This is in agreement with the CIN data (Fig. 4b) and with the notion of a cluster of CIN tumors with high NF-YAl/NF-YAs ratios being inserted in the EMT subtype of ACRG (Fig. 5c): this could be responsible for the correlation seen in EMT, but not in GS. To substantiate this point, we calculated the distribution of the NF-YAl/NF-YAs ratios in GS and EMT: Fig. 6b shows that GS has a flatter distribution, with more samples with very high ratios (35% are ≥ 1), whereas EMT has fewer samples with high ratios (25% are ≥ 1), but a larger population with ratios between 0.2 and 0.5. Thus, EMT is in part fed by the CIN samples that show high ratios (Fig S6b). Note that EBV and MSI have essentially no samples above a 0.35 ratio. Thereafter, we stratified EMT samples according to low and intermediate/high ratios: the curve of the latter significantly correlates to a worst outcome (p value 0.012) (Fig. 6c, Left Panel). In addition, we reasoned that the overall levels of NF-YAs might also be impactful: stratification according to NF-YAs levels indeed indicates a protective effect of this isoform (Fig. 6c, Right Panel). Finally, analysis on the levels of HFD subunits in ACRG subtypes yielded negative results (Fig. S7), except for NF-YB, whose high levels are protective in MSS;TP53^+^ (Fig. 6d). Altogether, these data reinforce the role of the relative levels of the two NF-YA isoforms in the outcome of EMT, as well as pointing at a novel role of NF-YB in the MSS;TP53^+^ subtype.

### NF-YAl is predominant in Claudin^low^ STAD tumors

**Figure 7:**
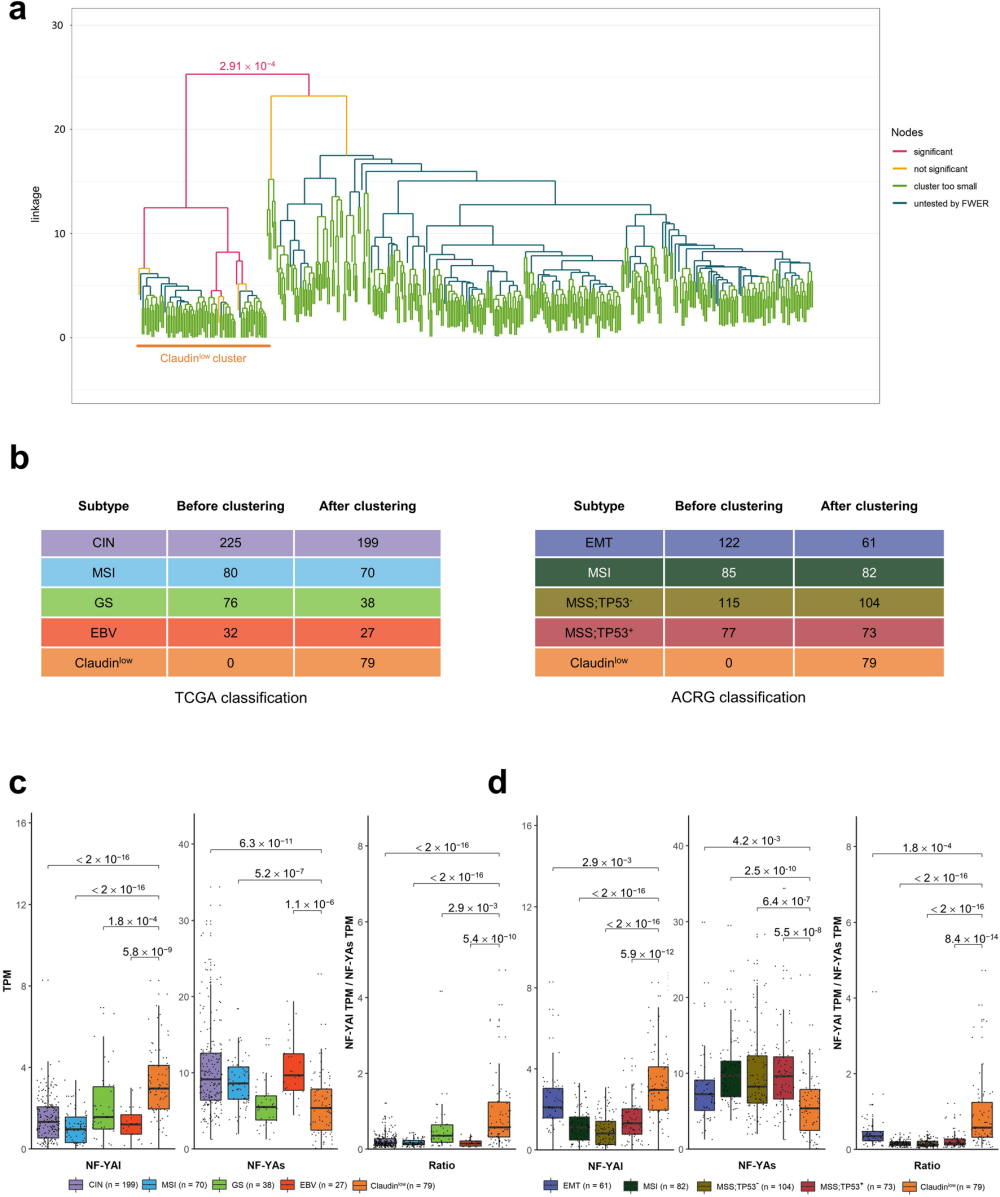
NF-YAl is predominant in Claudin^low^ STAD tumors. (**a**) Hierarchical clustering of all TCGA tumor samples according to the 24 genes signature described in Ref. ^37^. (**b**) Sample repartition in the TCGA (Left Panel) and ACRG (Right Panel) classifications, including the new Claudin^low^ group identified in (**a**), before and after the clustering procedure. (**c**) Box plots of expression levels of NF-YAl (Left Panel), NF-YAs (Central Panel), and NF-YAl/NF-YAs ratio (Right Panel) in the TCGA classification, including the Claudin^low^ group identified in (**a**). (**d**) Same as (**c**), except that the NF-YA isoforms expression levels are calculated for the ACRG classification subtypes. p values are calculated using the Wilcoxon rank-sum test.

We previously reported on association of high NF-YAl levels in a subclass of BRCA showing low levels of Claudin 3/4/7 expression^25^, a cluster associated with EMT features and poor prognosis. By analyzing TCGA STAD data, Nishijima et al. identified a specific group of tumors—46 samples—based on three features: epithelial to mesenchymal transition (EMT), tumor-initiating cells (TIC) and a Claudin^low^ phenotype^37^; this group was separated from CIN and GS (TCGA classification) and EMT (ACRG classification). Importantly, these Authors derived a 24-strong gene signature predictive of this subclass: we used it to conduct a hierarchical clustering of the entire TCGA dataset; Fig. 7a shows the dendrogram with the identification of 79 samples with these gene expression features; this cohort is clearly separated by the other tumors based on a strong statistical bias (p value: 2.91 × 10^–4^). We first checked how this signature features each subtypes: Fig. S8 shows below zero median Z scores of CIN, EBV and MSI (TCGA), MSI, MSS;TP53^-^ and MSS;TP53^+^ (ACRG); instead, good concordance is scored within the GS and EMT groups. Because of the presence of low levels of epithelial Claudins, we will refer to this group as Claudin^low^. Next, we positioned this group within the other TCGA and ACRG subtypes (Fig. 7b): most tumors of the Claudin^low^ cluster are from the GS and CIN (TCGA) and EMT (ACRG) subtypes. In essence, the Claudin^low^ group could be classified as new within TCGA, while being essentially a subclass of the EMT ACRG subtype. Overall, these data confirm the existence of the subgroup proposed by Nishijima et al., further expanding it to 79 TCGA samples, with robust statistical significance.

Next, we evaluated the expression of NF-YA isoforms and their relative ratio including the Claudin^low^ group. Figure 7c,d show the results according to the TCGA and ACRG subtypes, respectively: NF-YAl is mostly present in the Claudin^low^ class, with far lower levels in the remaining samples of the ACRG EMT subtype. On the contrary, NF-YAs is lowest in Claudin^low^, and higher in all other ACRG and TCGA subtypes, with the exception of GS. As a consequence, the NF-YAl/NF-YAs ratio is significantly increased (lowest p values: 10^–16^) mostly in the Claudin^low^ group. These data indicate that NF-YAl is mostly associated to a discrete number of STAD samples with EMT and Claudin^low^ features.

To verify the overlap between the Claudin^low^ and NF-YAl^high^ (and NF-YAs^low^) subsets, we stratified the clinical outcome of Claudin^low^ tumors according to NF-YA isoforms expression (High, Intermediate, Low): no further worsening of prognosis in PFI curves is scored according to the different levels of NF-YA isoforms (Fig. S9, Upper Panels), nor NF-YAl/NF-YAs ratio (Fig. S9, Lower Panel). We conclude that there is a large overlap between the subset classified as Claudin^low^ and NF-YAl^high^ tumors.

### CCAAT box is enriched in upregulated pathways of Claudin^low^ samples

**Figure 8:**
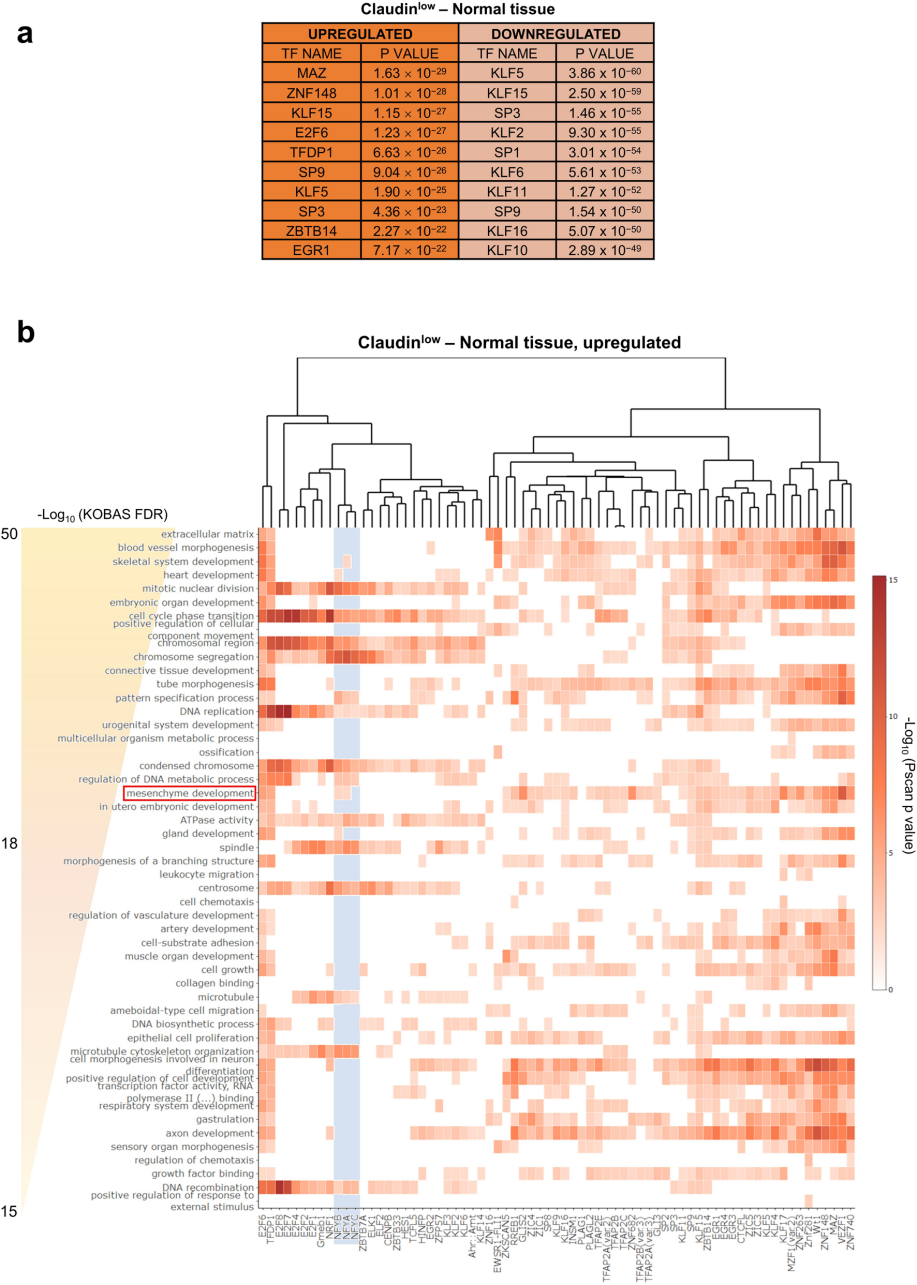
DEG in Claudin^low^ subtype. (**a**) List of enriched TFBS in promoters of upregulated and downregulated genes in the Claudin^low^ subtype, compared to normal tissue, as predicted by Pscan. (**b**) Heatmap showing the p values, as computed by Pscan, associated to the enrichment in multiple TFBS motifs (columns) of the top 50 most represented GO terms (rows) among upregulated genes in Claudin^low^ samples, as emerged from the KOBAS analysis. The light blue area highlights NF-Y subunits binding site motifs (CCAAT box). Only terms with less than 500 background genes and TFBS with a significant enrichment in more than 10 terms (p < 0.01) were included in the heatmap.

To further investigate the Claudin^low^ cluster, we compared pathways in Claudin^low^ and EMT versus normal samples. The analysis of DEG in EMT shows absence of CCAAT in promoters (Fig. S10a). Across EMT upregulated pathways, we did find mesenchymal terms such as *extracellular matrix*, *heart development*, *mesenchyme development* (Fig. S10b). Within the TF motifs enriched in the promoters of genes of each single category, we observed significant enrichment of the NF-Y motif in *cell-cycle* terms, as expected, and in *mesenchyme development* and *pattern specification process*. In downregulated pathways, we observed different *metabolism* terms, also expected (Fig. S10c). The same analysis performed on Claudin^low^ samples did not yield NF-Y motifs as enriched in deregulated genes, but rather MAZ, E2F6 and KLFs motifs (Fig. 8a); these TFs were confirmed by analyzing ChIP-seq data from the ChIP-Atlas database^38^ (Supplementary Table S3). Among upregulated pathways we found *extracellular matrix* and *mesenchyme development* terms (*heart development*, *skeletal system*, and *pattern specification process*). As above, the CCAAT box was enriched in terms related to mesenchyme (Fig. 8b). Various metabolic processes populated the downregulated pathways (fatty acid and lipid metabolic process), expectedly regulated by NF-Y and with CCAAT motifs (Fig. S11).

## Discussion

Because of its histone-like structure^17^, positioning within promoters^16^, synergistic connections with many other TFs and interactions with coactivators, NF-Y is believed to play a pioneering role in “opening” promoter structures and correct positioning of RNA Pol II^39^. Specifically, NF-Y is important for genes required for cell proliferation^20^. We describe here an investigation on NF-Y subunits levels in gastric cancer. We report the presence of CCAAT in commonly overexpressed genes and overexpression of NF-YA isoforms, as well as a prognostic value of their relative levels. We also report on overexpression of the HFD subunits, and clinical significance of NF-YB.

CCAAT boxes have been routinely found in promoters of genes overexpressed in cancer, first in large microarrays profiling^15^ and more recently in RNA-seq datasets. Our analysis of TCGA identified CCAAT in overexpressed genes, typically with E2Fs sites, in line with the pro-growth role of these TFs. Specifically, two schemes are starting to emerge. In the first, CCAAT is enriched globally, and indeed at the top of the TFBS list, when all upregulated genes are computed: it is the case of lung tumors^26,27^; in the second, the enrichment is found either in specific subtypes—iCluster 3 in HCC^28^—or only in DEG shared by all subtypes, as in BRCA^25^ and STAD, as shown here. In global STAD DEG, TFBS in promoters of upregulated genes contain the familiar E2Fs motifs, along with Zn Finger TFBS (SPs/KLFs), but CCAAT is absent. As in BRCA, however, it comes out first when considering the core group of upregulated genes shared in all STAD subtypes. We also find that CCAAT is absent in promoters of genes downregulated in STAD, as for all other types of cancer examined so far. This further reinstates that this element is not a “general” signal enriched in promoters per se, but rather a core logo driving expression of genes associated to growth, not necessarily related to transcriptional features that are cancer- or subtype-specific.

The HFD subunits are overexpressed in STAD, unlike in lung and breast tumors. We recently reported a similar scenario in HCC, in which high levels of these subunits correlate with worst prognosis in a specific subtype, iCluster1. In STAD, global or subtype-specific PFI curves are globally superimposable based on NF-YB or NF-YC expression, with one notable exception: the MSS;TP53^+^ ACRG subtype, in which high NF-YB levels correlate with a better prognosis. As for HCC, the fraction of p53 wt tumors in STAD is much higher—51%—than in other epithelial cancers (lung for example), in which the vast majority are p53 mutated, rendering comparisons with wt p53 samples essentially impossible. Note that the protective role of NF-YB in STAD is opposite to what we reported in HCC iCluster1 tumors, generally associated to wt p53 status: although direct NF-Y/p53 interactions have been reported in several studies^20^, the reasons for association of NF-YB levels to such genetic background is unclear. Nevertheless, a role of HFD subunits in cancer progression is starting to emerge; in this respect, measurement of protein levels in tumors deserve a close look in the future: in BRCA cell lines, for example, the NF-YB protein seems to be more variable than one could anticipate from mRNA levels^25^.

Overexpression of NF-YA mRNA is as obvious in STAD as in the tumors previously analyzed. Note that analysis of 22 cancer specimens confirms that higher expression is also found at the protein level^30^. In the same study, high levels of NF-YA and Cyclin E in TCGA STAD samples were associated to worsening of patients’ prognosis: yet, we do not find here a prognostic value of global levels of NF-YA. In another study, NF-YA high expression correlated with prognosis in a separate set of tumor samples analyzed by microarray profilings^31^, but only in the Diffuse (DF), not in the Intestinal (IT) subtype (Lauren classification). We add a novel and relevant twist, in that isoform ratios—rather than global levels—are clinically important within subclasses of STAD.

The two major NF-YA splicing isoforms differ in the Gln-rich trans-activation domain (TAD): NF-YAl has 28/29 extra amino acids coded by exon 3, predicted to impart different activation potential, as reported in mESCs and myoblasts^40,41^. In addition, a shorter isoform—NF-YAx—lacking sequences of exon-3 and exon-5 was recently found overexpressed in Neuroblastomas^42^. As in the other epithelial cancers, we find that NF-YAs predominates, but higher expression of NF-YAl, alone or coupled to lower levels of NF-YAs, is clinically relevant. The TCGA GS subtype is enriched in DF samples^8^, which is indeed in line with the data reported by Cao et al.^30^. GS tumors are characterized by earlier onset and expression of “cell adhesion” signatures. The NF-YAl/NF-Ys ratio is shifted in GS and the same pattern is observed stratifying tumors according to the ACRG classification: higher NF-YAl/NF-YAs ratios are found in EMT tumors. The relatedness of these subtypes in the two classifications was commented before^11–13^: indeed analysis of GO terms and pathways of DEG in these subtypes are in agreement with a mesenchymal phenotype. The ACRG EMT has 48 samples catalogued as CIN by TCGA: interestingly, the PFI of CIN patients indicates a worst prognosis following the NF-YAl/NF-YAs ratios.

Our comparative analysis of the whole set of TCGA tumors suggest clinical relevance for NF-YB and NF-YA isoforms in subgroups of the ACRG classification. Specifically, NF-YA-wise, the ACRG EMT group is more revealing than the TCGA GS, most likely because of the inclusion of CIN tumors with EMT-like profilings. While in the EMT group the role of NF-YA ratios is clinically visible, in the TCGA GS it is not. One possible explanation is the lower dispersion of ratios and lower number of samples in this latter group, making comparison of quartiles difficult. Incidentally, this also allowed to score a protective role of NF-YAs, completely missed by adhering to the TCGA classification. Another feature emerging in the ACRG classification is the protective role of high NF-YB levels, as discussed above. These differences might reflect the fact that RNA profilings are the basis of ACRG, while TCGA factored in other genetic and epigenetic features of STAD.

The parallel of the present data with what we found in breast carcinoma is noteworthy. NF-YAs is also predominant in BRCA, except in the Claudin^low^ subset of Basal-like tumors, that have higher levels of NF-YAl. This is associated to a shift in DEG in these tumors, from signatures dominated by proliferative terms in NF-YAs^high^ tumors, toward activation of EMT signatures. In turn, this is clinically associated to an aggressive, metastatic, drug-resistant behavior. As in BRCA, the NF-YAl/NF-YAs ratio is clinically informative in STAD, but in this case the protective role of NF-YAs^high^ in the EMT subtype is novel. Nishijima et al. showed that overall survival curves and Hazard ratios of the 46 Claudin^low^ patients are indeed worse with respect to other subtypes, dramatically so within the ACRG-classified patients. This suggests that the Claudin^low^ partitioning is particularly significant with ACRG. We extended this group to 79 TCGA tumors by using the signature described: our results confirm and extend the scenario proposed by these Authors, particularly within the ACRG classification, which better partitions the protective role of NF-YAs from the detrimental role of NF-YAl in the Claudin^low^ group. Furthermore, it appears manifest the overlap of tumors with Claudin^low^ and NF-YAl^high^ features.

In general, these data invite further analysis in epithelial cancers to identify (i) Claudin^low^ signatures in other types of epithelial cancers, and (ii) a threshold of NF-YA isoforms ratios, rather than overall levels, possibly responsible for shifting DEG away from proliferative, cell cycle genes toward mesenchymal ones.

## Materials and methods

### RNA-seq datasets

As of December 2020, there were RNA-seq data on 415 STAD primary tumors in TCGA and 35 non-tumor tissues. We downloaded the corresponding RSEM scaled count data from the http://firebrowse.org/ web page. The last published classification of STAD samples in the four molecular subtypes made by TCGA referred to 387 of the 415 tumors, and we retrieved it from the https://www.cbioportal.org/ web page^43,44^; a different classification was proposed by ACRG on 204 TCGA tumors^9^. All the experiments involving human data in these public datasets adhered to relevant ethical guidelines. The DeepCC tool^35^ was used to classify RNA-seq dataset of all tumors in TCGA, according to the TCGA and ACRG classification, using as a training set the tumors already classified by TCGA and ACRG, respectively.

We retrieved the FASTQ files associated to the 37 CCLE stomach cell lines (accession code: PRJNA523380)^45^, as well as the 29 cell lines collected by Lee et al. (accession code: PRJNA327709)^34^, using the SRA Explorer website (https://sra-explorer.info/). From the FASTQ files, we calculated mRNA expression with RSEM-1.3.3.

### Gene expression analysis

Differential gene expression analysis of RNA-seq data was performed using R package *DESeq2*^46^. The Tumor versus Normal expression fold change (FC) denotes upregulation or downregulation according to the FC value. Log_2_FC, and the corresponding false discovery rate (FDR), were reported by the R package. FDR < 0.01 and |log_2_FC|> 0.5 were set as inclusion criteria for DEG selection in tumor/subtype versus normal samples.

### Gene ontology, pathway enrichment and transcription factor binding site analysis

We used KOBAS 3.0 (http://kobas.cbi.pku.edu.cn/anno_iden.php) for pathway enrichment analysis using the ENTREZ gene IDs. The TFBS and de novo motif analyses were performed using the Pscan software^36^, while ChIP-seq experiments enrichment analyses were conducted with ChIP-Atlas^38^. To obtain TFBS enrichment heatmaps, input genes collections of the top GO terms from KOBAS analysis, sorted by FDR, were analyzed individually with Pscan. Only GO terms with less than 500 background genes were included, and TFBS motif enriched (Pscan p value < 0.01) in less than 10 terms were filtered out.

### Analysis of clinical data

We retrieved clinical data related to the TCGA STAD samples and progression free interval—PFI—time records of patients, respectively, from the https://www.cbioportal.org/ and the http://xena.ucsc.edu/ web pages^43,44,47^. We stratified all the tumors for which PFI records were available according to NF-Y subunits expression at gene level, NF-YA isoforms expression, and NF-YAl/NF-YAs ratio, into three groups (Low = first quartile, Intermediate = second and third quartiles, High = fourth quartile). Survival analysis was performed according to the Kaplan–Meier analysis and log-rank test^48^.

### Hierarchical clustering and Z scores computation

TCGA samples RSEM scaled count data were converted into TPM, log_2_-transformed, and median centered; we then performed a hierarchical clustering of the samples with the R package *SigClust2* (version 1.2.4) with “average” linkage and “euclidean” metric options, while the alpha parameter was set to 0.05. Daughter nodes were tested if significance was achieved at the corresponding parent node, according to the built-in FWER controlling procedure. We obtained Z scores from log_2_-transformed expression data for each gene of the Claudin^low^ signature, and a median Z score for each sample was computed across the genes of the signature.

### Statistical analysis

Analyses were performed in the R programming environment (version 4.0.3), with the *ggplot2*, *ggpubr*, *survival*, *survminer, tidyverse* packages. Single comparisons between two groups were performed with the Wilcoxon rank-sum test.

## Supplementary Information

Supplementary Figure S1.

Supplementary Figure S2.

Supplementary Figure S3.

Supplementary Figure S4.

Supplementary Figure S5.

Supplementary Figure S6.

Supplementary Figure S7.

Supplementary Figure S8.

Supplementary Figure S9.

Supplementary Figure S10.

Supplementary Figure S11.

Supplementary Table S1.

Supplementary Table S2.

Supplementary Table S3.

## Abbreviations

TCGA: The Cancer Genome Atlas
ACRG: Asian Cancer Research Group
NF-YAl: Nuclear factor Y subunit A isoform long
NF-YAs: Nuclear factor Y subunit A isoform short
NF-YB: Nuclear factor Y subunit B
NF-YC: Nuclear factor Y subunit C
E2F: E2 factor
TF: Transcription factor
TFBS: Transcription factors binding sites
FDR: False discovery rate
HFD: Histone fold domain
STAD: Stomach adenocarcinoma
BRCA: Breast carcinoma
LUSC: Lung squamous cells carcinoma
LUAD: Lung adenocarcinoma
HCC: Hepatocellular carcinoma
HNSCC: Head and neck squamous cells carcinoma
CIN: Chromosome instability
EBV: Epstein-Barr virus
GS: Genomically stable
MSI: MicroSatellite instability
EMT: Endothelial to mesenchymal transition
MSS: MicroSatellite stable
TP53: Tumor protein 53
TIC: Tumor-initiating cells
DEG: Differentially expressed genes
PFI: Progression free interval
