# Supplementary figures and images for: NF-Y subunits overexpression in gastric adenocarcinomas (STAD)
Source: Sci Rep. 2021 Dec 9;11:23764. doi: 10.1038/s41598-021-03027-y (PMC8660849; doi:10.1038/s41598-021-03027-y)

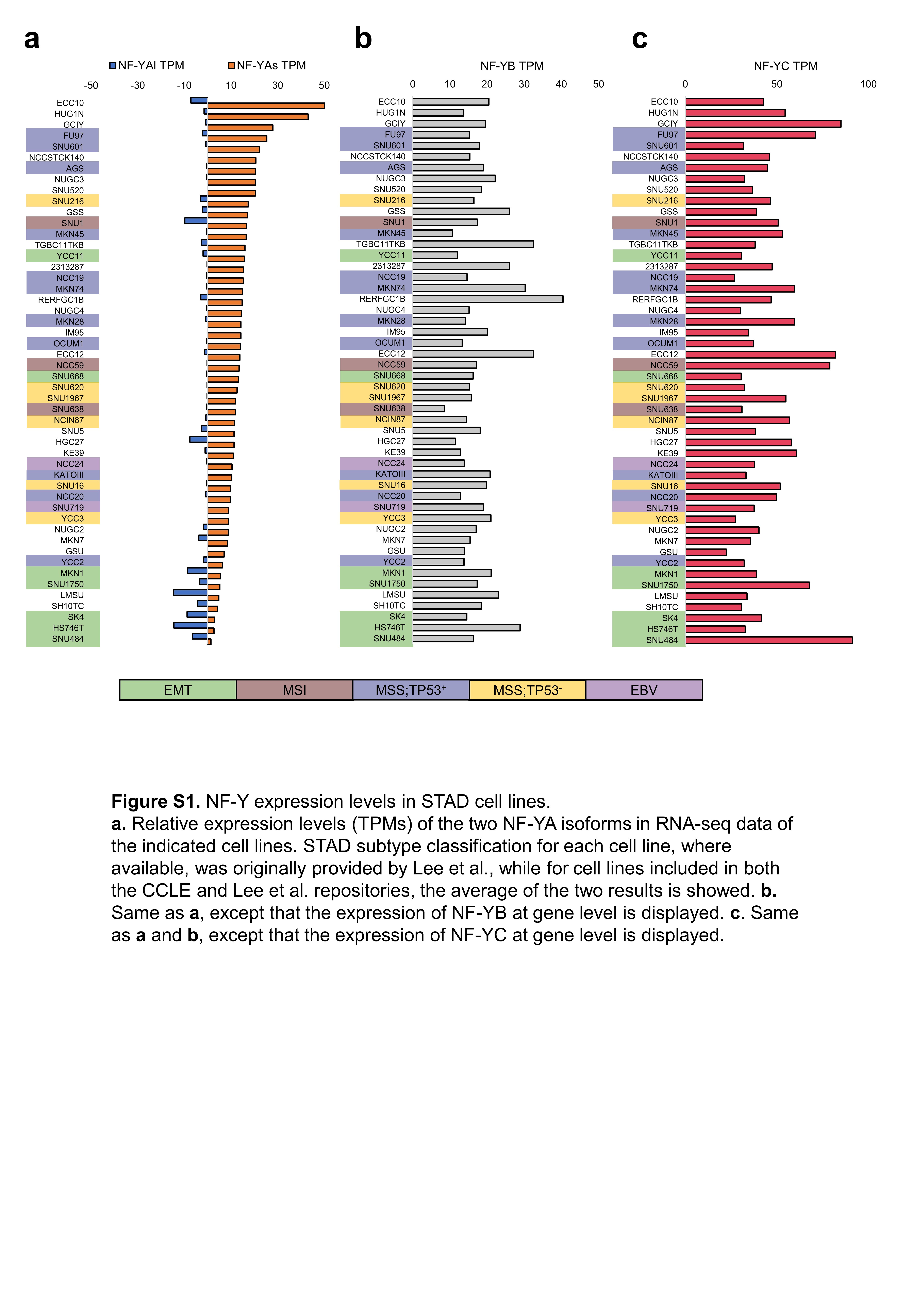

Supplement: Supplementary file 1 — Supplementary Figure S1. [file 41598_2021_3027_MOESM1_ESM.jpg]

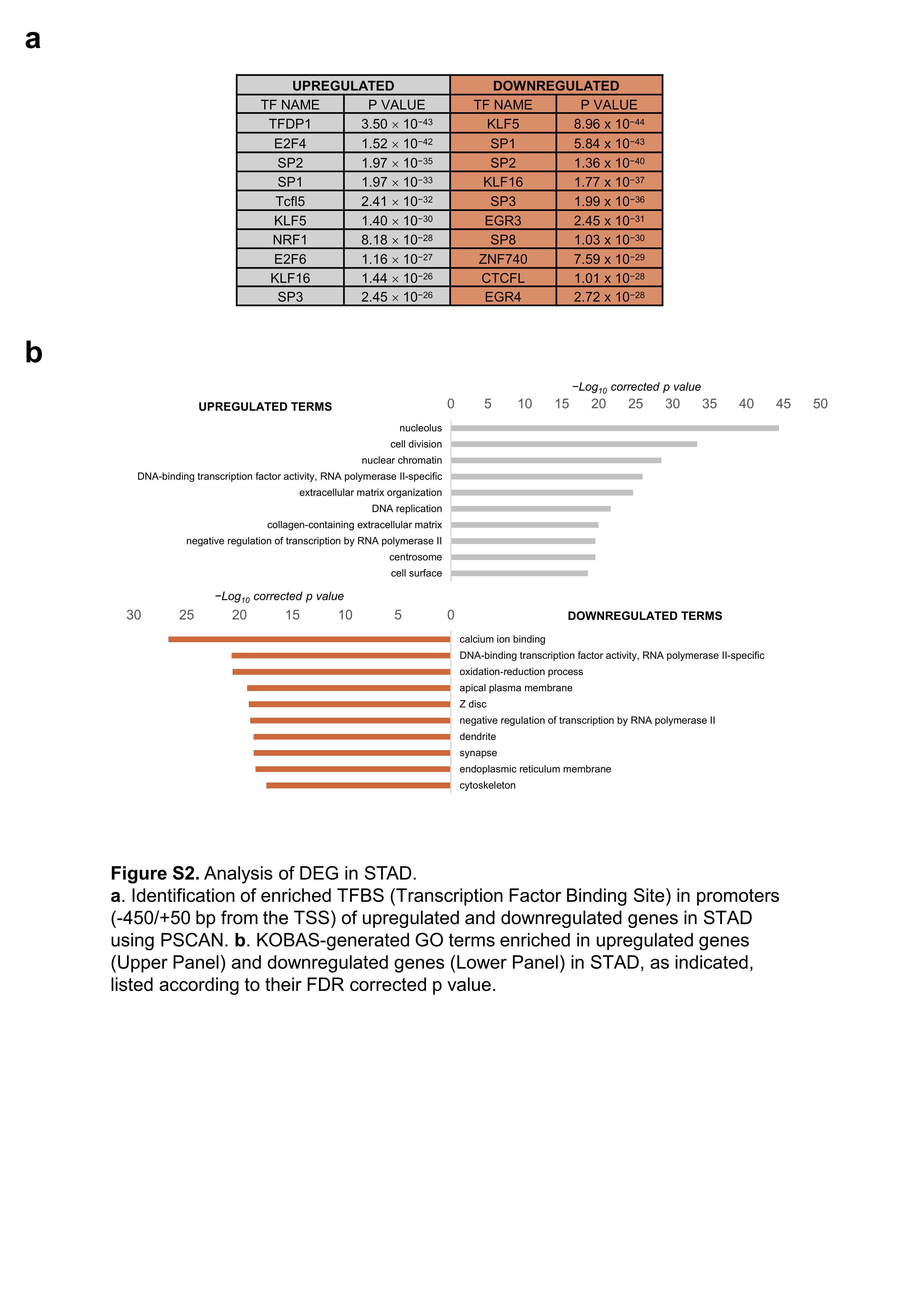

Supplement: Supplementary file 2 — Supplementary Figure S2. [file 41598_2021_3027_MOESM2_ESM.jpg]

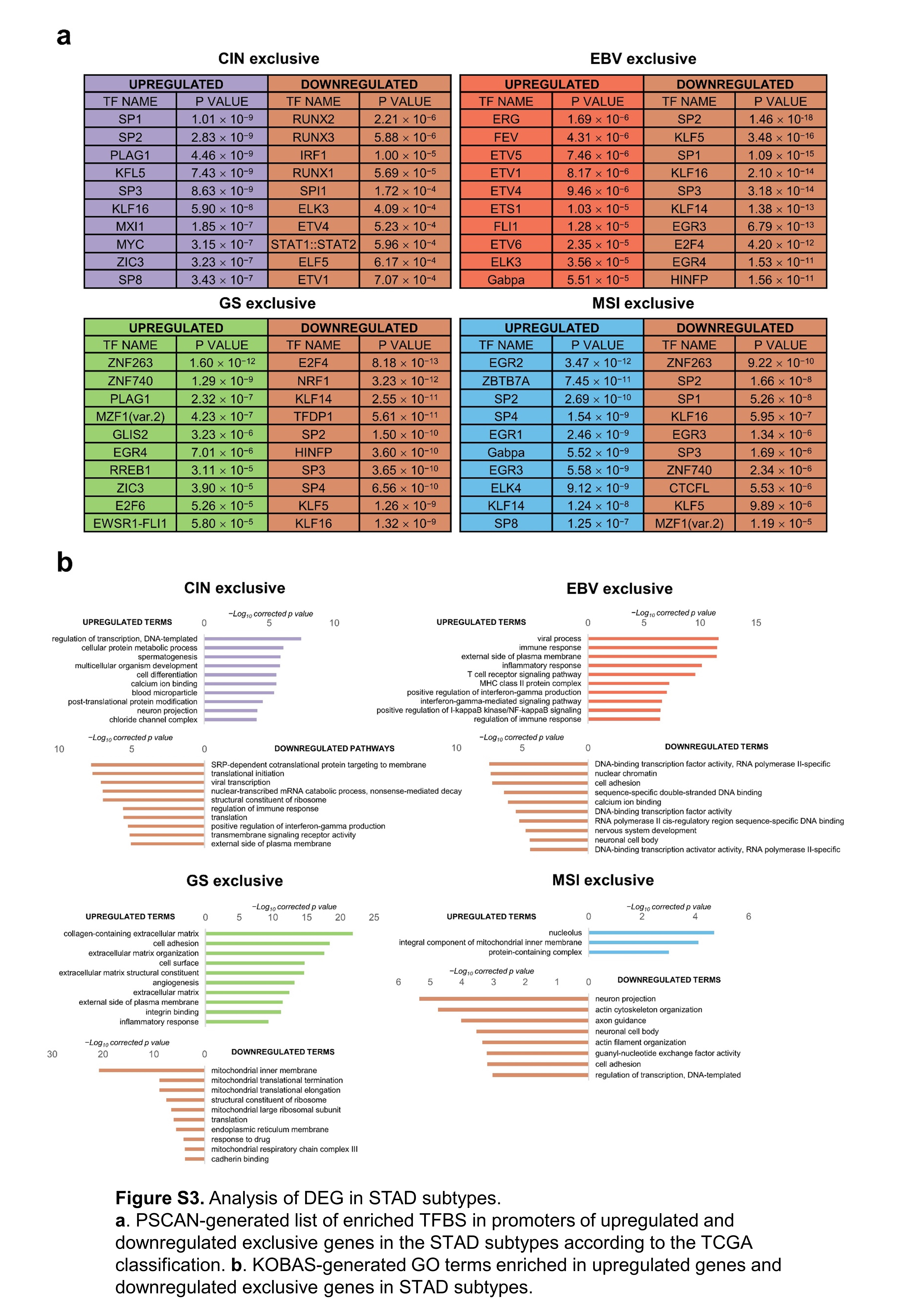

Supplement: Supplementary file 3 — Supplementary Figure S3. [file 41598_2021_3027_MOESM3_ESM.jpg]

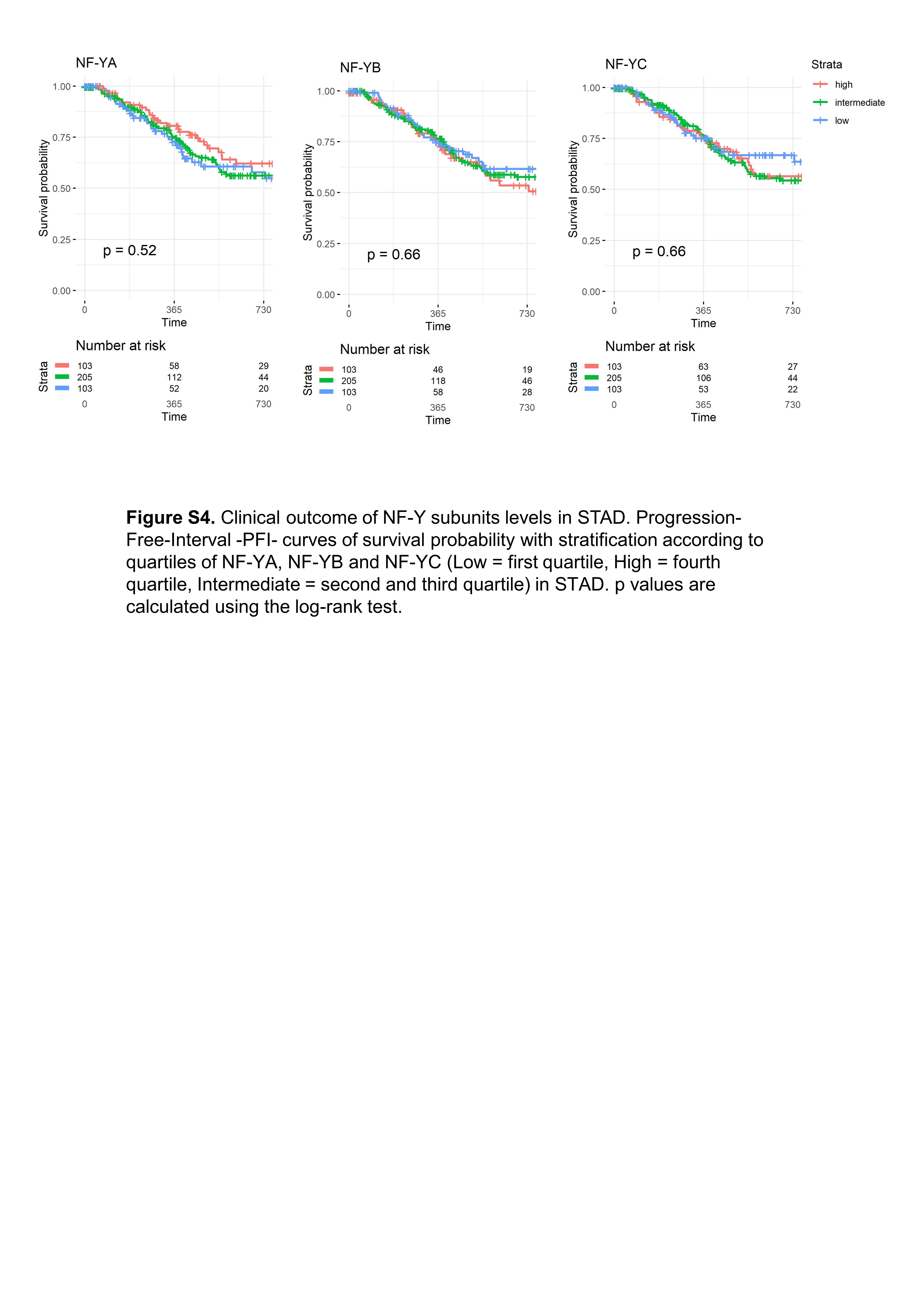

Supplement: Supplementary file 4 — Supplementary Figure S4. [file 41598_2021_3027_MOESM4_ESM.jpg]

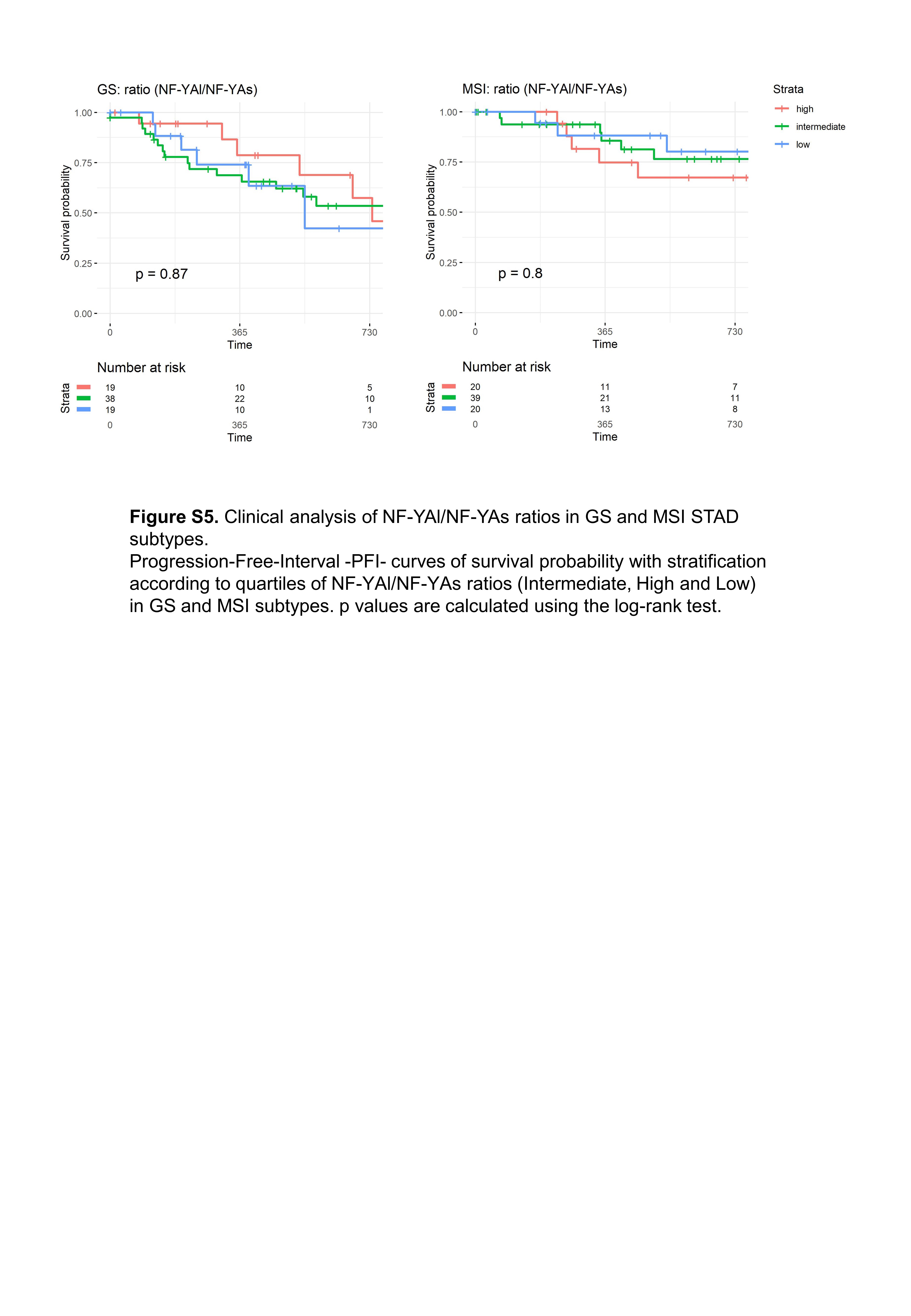

Supplement: Supplementary file 5 — Supplementary Figure S5. [file 41598_2021_3027_MOESM5_ESM.jpg]

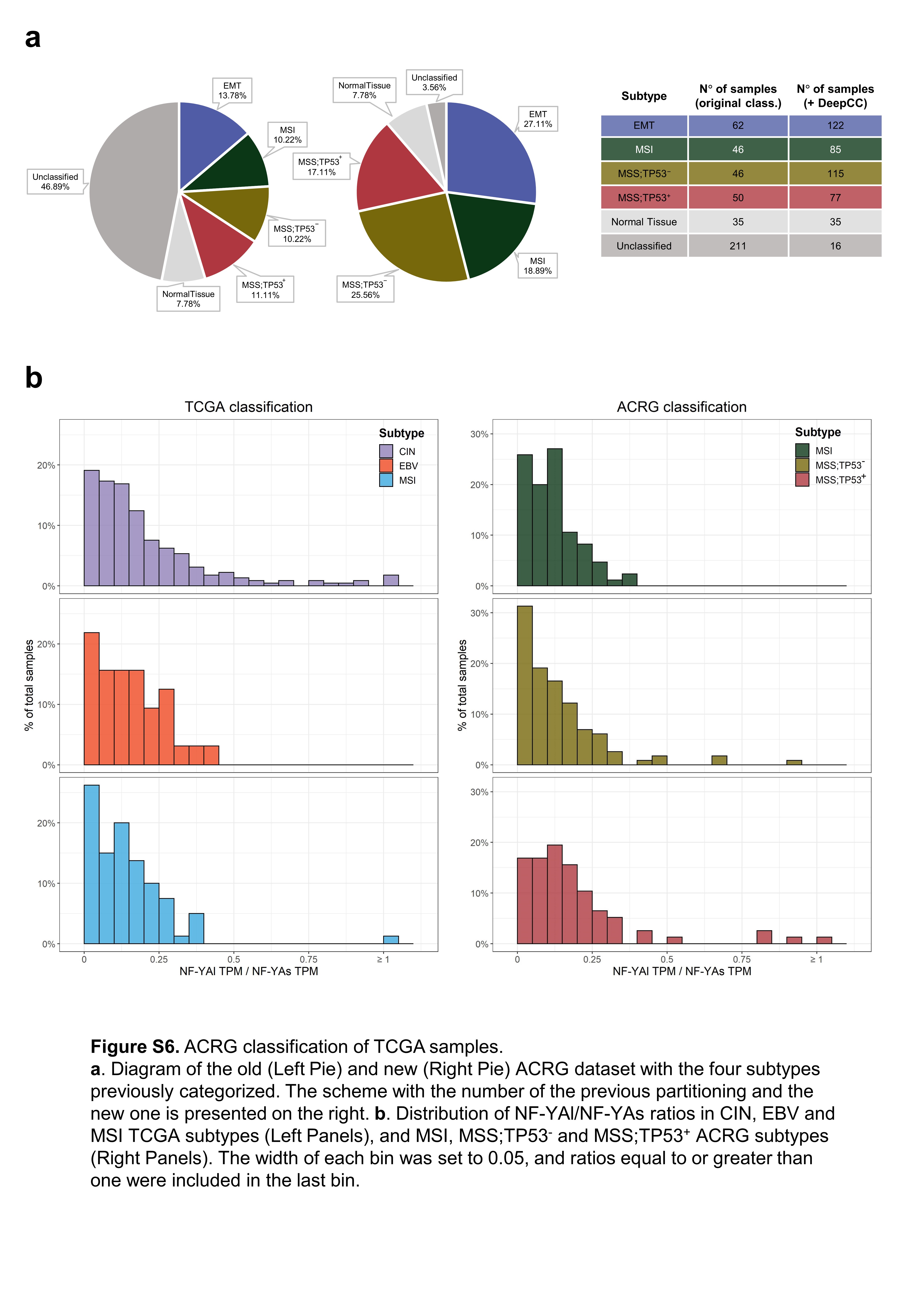

Supplement: Supplementary file 6 — Supplementary Figure S6. [file 41598_2021_3027_MOESM6_ESM.jpg]

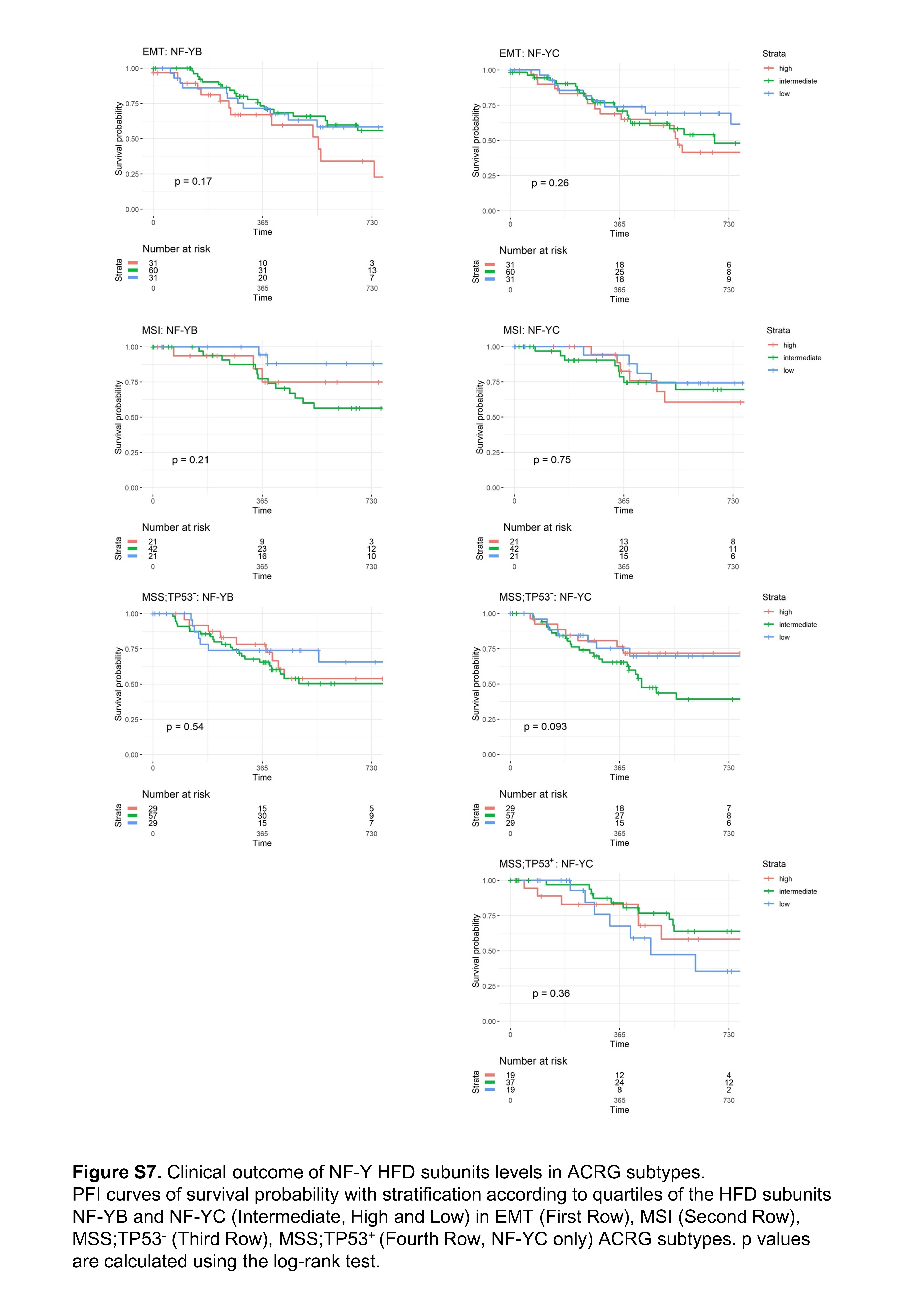

Supplement: Supplementary file 7 — Supplementary Figure S7. [file 41598_2021_3027_MOESM7_ESM.jpg]

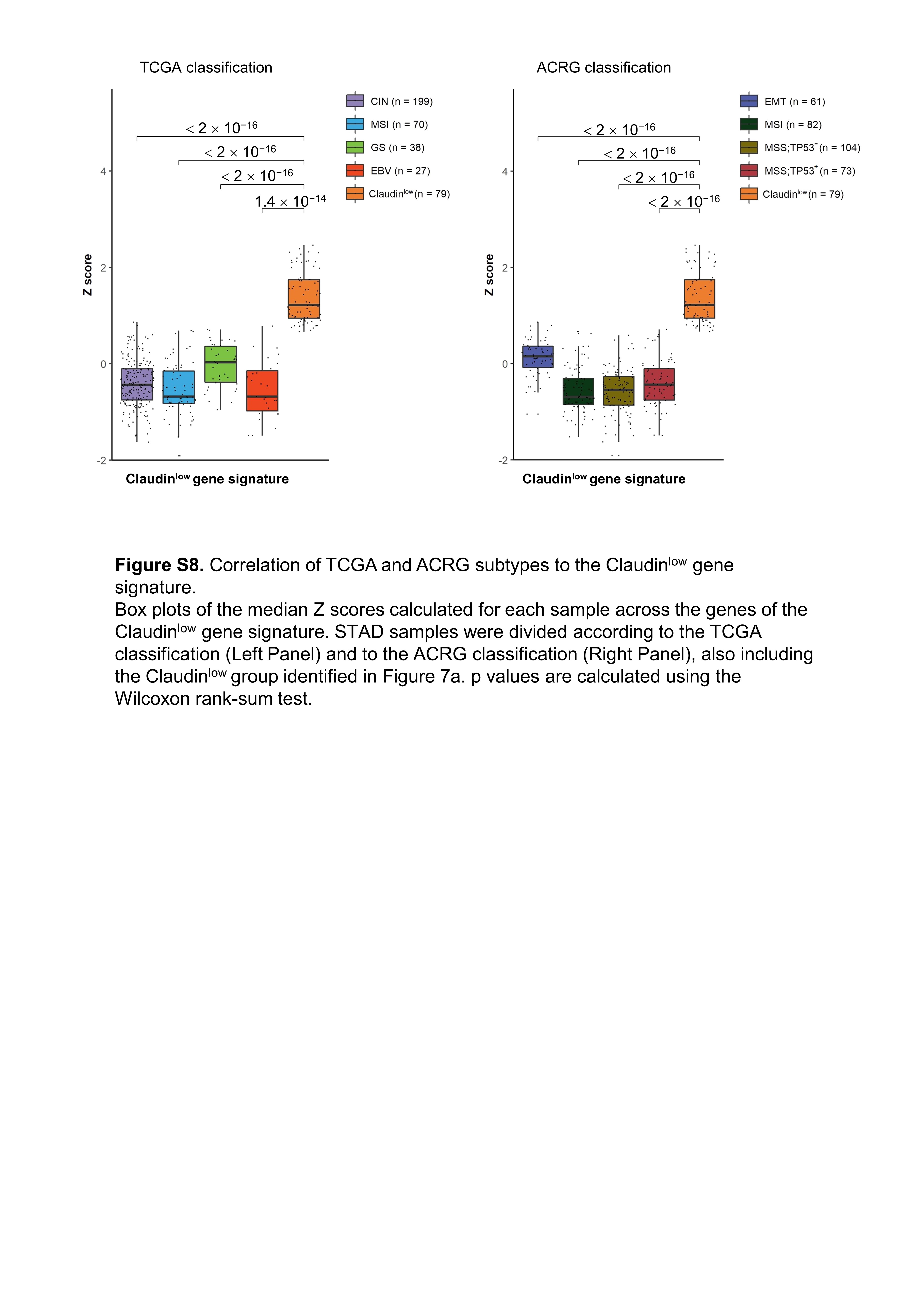

Supplement: Supplementary file 8 — Supplementary Figure S8. [file 41598_2021_3027_MOESM8_ESM.jpg]

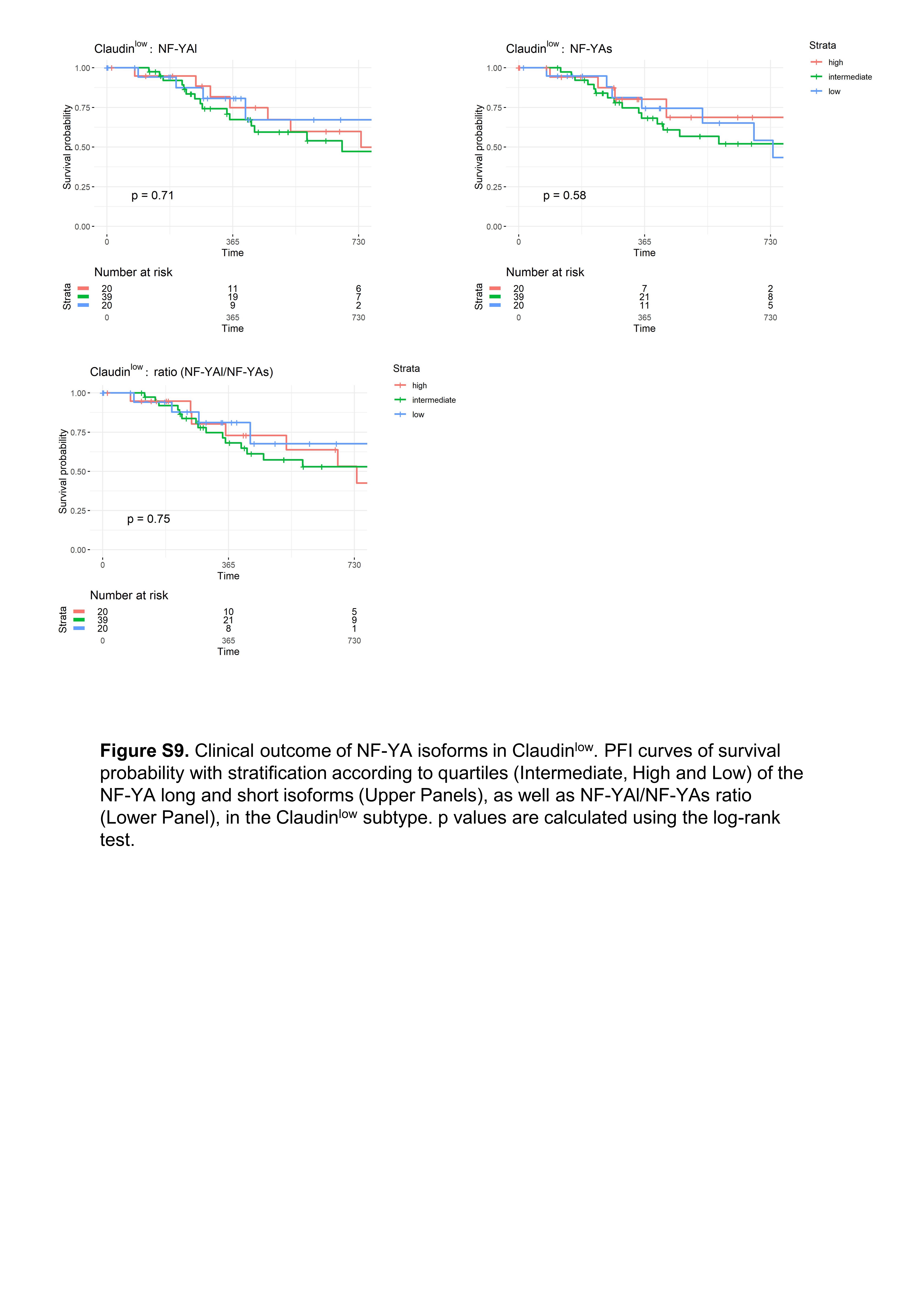

Supplement: Supplementary file 9 — Supplementary Figure S9. [file 41598_2021_3027_MOESM9_ESM.jpg]

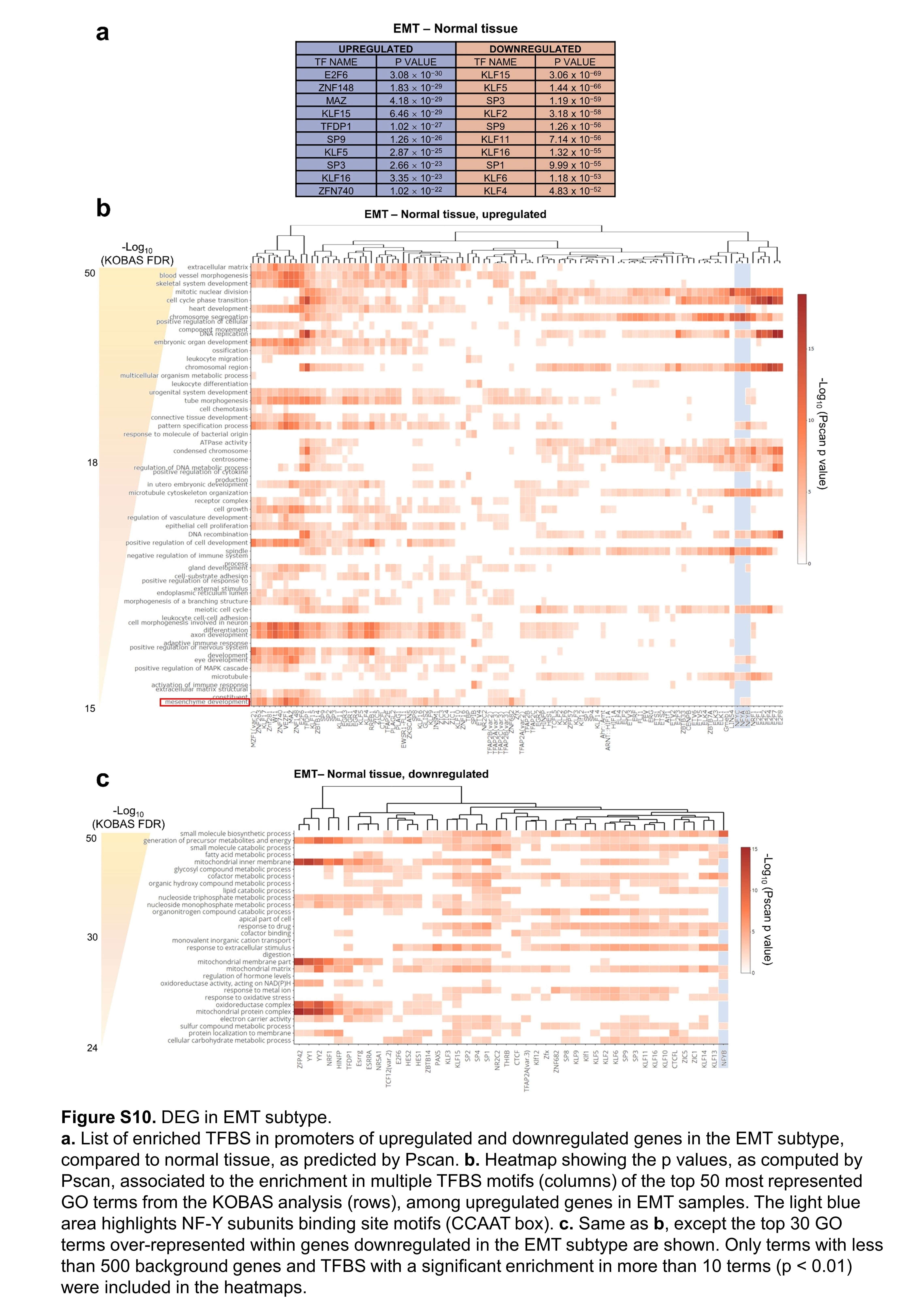

Supplement: Supplementary file 10 — Supplementary Figure S10. [file 41598_2021_3027_MOESM10_ESM.jpg]

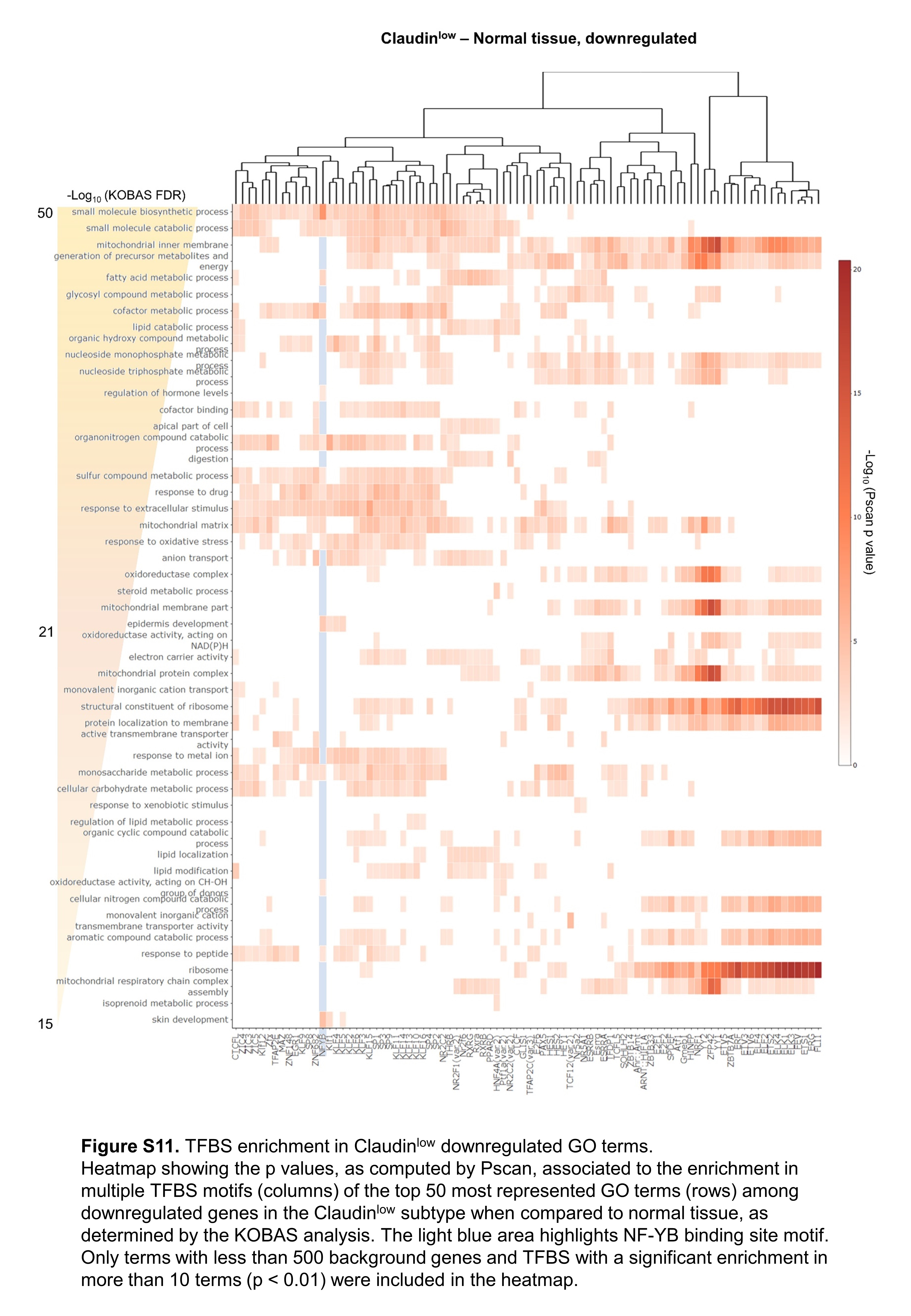

Supplement: Supplementary file 11 — Supplementary Figure S11. [file 41598_2021_3027_MOESM11_ESM.jpg]
